# Nitrate of Silver in Dyspepsia or Chronic Gastritis

**Published:** 1853-06

**Authors:** W. A. Gillespie

**Affiliations:** Louisa Co., Va.


From the Boston Med. and Surg. Jour.

*Nitrate of Silver in Dyspepsia or Chronic Gastritis.*

Sir,—During the last twenty years I have had many cases of inveterate dyspepsia or chronic gastritis. A long course of dieting, with occasional *pro re nata* medicines, has afforded partial relief only, in many cases. Some years ago, I used nitrate of silver and opium in cases attended by chronic diarrhœa, with success, and more recently I have used the same remedy when diarrhœa was only occasional and often absent. From the trials I have made and the success I have obtained, I think the nitrate promises to be a very useful remedy. My experience has not been large in the use of it as a remedy for dyspepsia ; but in a few cases, and so far as I have made trials, it has acted with the promptness and certainty of quinine in ague. I begin with a pill containing a quarter of a grain of nitrate of silver and a quarter of a grain of opium, administered three times a-day. After using this quantity several days, I double the quantity of the nitrate, but not of the opium; and when opium is contra-indicated, I omit it altogether. In some cases I gradually increase the nitrate to one grain (and even two grains in one instance) three times a-day. The remedy certainly deserves further trial, and my design in this short communication is to call the attention of the profession to an important subject, and to request others to try the remedy and note the results.

W. A. GILLESPIE, M. D.

Louisa Co., Va., March 24, 1853.

